# Drosophila Enhancer of Rudimentary Homolog, ERH, Is a Binding Partner of RPS3, RPL19, and DDIT4, Suggesting a Mechanism for the Nuclear Localization of ERH

**DOI:** 10.1155/2016/8371819

**Published:** 2016-10-18

**Authors:** Stuart I. Tsubota, Anthony C. Phillips

**Affiliations:** ^1^Department of Biology, The College at Brockport, Brockport, NY 14420, USA; ^2^Department of Biology, Saint Louis University, St. Louis, MO 63103, USA

## Abstract

The protein enhancer of rudimentary homolog, ERH, is a small, highly conserved protein that has been found in animals, plants, and protists. Genetic and biochemical interactions have implicated ERH in the regulation of pyrimidine biosynthesis, DNA replication, transcription, mRNA splicing, cellular proliferation, tumorigenesis, and the Notch signaling pathway. In vertebrates and insects, ERH is nuclearly localized; however, an examination of the ERH amino-acid sequence does not reveal any nuclear localization signals. In this paper we show that the first 24 amino acids contain sequences necessary and sufficient for nuclear localization. Through yeast two-hybrid screens, three new binding partners of ERH, RPS3, RPL19, and DDIT4, were identified. RPS3 was isolated from both human and Drosophila screens. These interactions suggest functions of ERH in cell growth, cancer, and DNA repair. The ERH sequences necessary for the interactions between ERH and RPS3 and RPL19 are mapped onto the same 24-amino-acid region in ERH which are necessary for nuclear localization, suggesting that ERH is localizing to the nucleus through binding to one of its DNA-binding partners, such as RPS3 or RPL19.

## 1. Introduction

The Drosophila* enhancer of rudimentary*,* e(r)*, gene encodes enhancer of rudimentary homolog (ERH), a small protein of 104 amino acids [1, 2]. ERH is a highly conserved protein that has been found in plants, animals, and protists [2]. Drosophila ERH is 76% identical to the vertebrate ERH, 49% identical to the* C. elegans* ERH, and 40% identical to the plant (Arabidopsis) ERH. The vertebrate ERH homologues are very highly conserved. The human, mouse, and Xenopus proteins are identical and differ from the zebrafish ERH by a single conservative amino-acid change.

**Figure 1:**
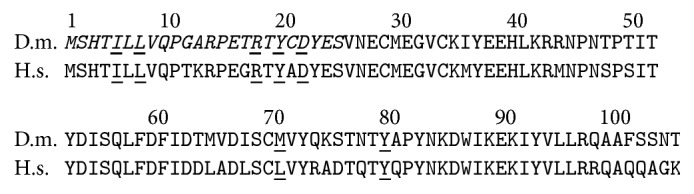
Comparison of the primary structures of the Drosophila and human ERH. The two proteins align perfectly without any gaps over their entire length. They are 76% identical. The positions of the seven amino acids that have been determined to be points of contact between the subunits of the ERH dimer are underlined. Six out of the seven are conserved between Drosophila and human, and the seventh (L to M) is a very conservative change. The N-terminal 24 amino acids that are necessary and sufficient for the nuclear localization of the Drosophila ERH and for the interactions with RPS3 and RPL19 are in italic.

The mouse and human proteins have been synthesized and purified from* E. coli *and the crystal structures determined [3, 4]. The secondary structure is very similar to the predicted secondary structure [2]. The protein contains three *α*-helices and a *β*-sheet that fold into a novel three-dimensional structure that comprises a single domain. The *β*-sheet is formed by four antiparallel *β*-strands. In vitro, human ERH exists as a dimer and the dimerization occurs via binding between the *β*-sheets of the two monomers. The binding is accomplished through primarily seven highly conserved amino acids (Figure 1).

A number of studies in vertebrates have indicated that ERH has a nuclear function. Yeast two-hybrid screens have identified possible nuclear partners for ERH. These include a transcription factor, DCoH/PCD in Xenopus [5], a protein involved in the regulation of DNA replication in humans, PDIP46/SKAR [6], and Ciz1, a nuclear zinc-finger protein that may regulate DNA synthesis and cyclin dependent kinases in humans [7, 8]. ERH has been shown to coimmunoprecipitate with SPT5, a transcription elongation factor [9], and with FCP1, a TFIIF-associating component of CTD phosphatase [10]. ERH has also been shown to interact with the spliceosome protein SNRPD3 and to be required for the splicing of the mRNA of CENP-E, a mitotic motor protein [11]. This diverse set of nuclear interactions suggests that ERH may be involved in a number of general nuclear functions involving DNA replication, transcription, and mRNA splicing. These studies also predict that ERH is nuclearly localized. This prediction is supported by the nuclear localization of an EGFP-ERH fusion protein in transfected NIH/3T3 cells [6]. Further studies with antibodies to Drosophila ERH showed that the endogenous ERH is localized to the nucleus in Drosophila Schneider cell lines and in cells of the early Drosophila embryo [12].

Searches for potential nuclear localization signals in Drosophila and human ERH by computer programs designed to identify nuclear localization signals (PredictProtein, WoLF PSORT, and cNLS mapper) fail to identify any. This presents the question as to how ERH is nuclearly localized. In this paper we define the sequence needed for the nuclear localization of ERH and describe the identification of new potential nuclear binding partners of ERH. The amino-acid sequences of ERH that are necessary for binding to its partners and for its nuclear localization are the same. This suggests that ERH is nuclearly localized through binding to these proteins or other binding partners.

## 2. Materials and Methods

### 2.1. The Yeast Two-Hybrid Interaction Trap and Interaction Assay

For these procedures, all of the yeast strains, plasmids, and cDNA prey libraries were obtained from the labs of Dr. Roger Brent while he was at Harvard University and Dr. Russ Finley (http://proteome.wayne.edu/index.html). Detailed procedures can be obtained from Dr. Finley's website. The host* Saccharomyces cerevisiae* strain EGY191 is a* his3*,* ura3*,* trp1*, and* leu2* auxotroph carrying a chromosomal insertion of LexA binding sites upstream of the endogenous* LEU2* gene. This strain also contained the plasmid pRB1840, which contains LexA DNA-binding sites in front of a* LACZ* reporter gene. EGY191/pRB1840 was then transformed with the bait plasmid pEG202 containing the appropriate LexA-*e(r)* fusion gene to be used in the interaction-trap screens or the interaction assays.

### 2.2. Full-Length Human and Drosophila ERH as the Bait

The* D. melanogaster e(r)* coding region was amplified from an* e(r)* cDNA via PCR using the primers 5′ GGGGATCCCCATGTCGCACACCATCCT 3′ and 5′ GGCTCGAGTTAGGTATTG GAACTAA 3′ (Table 1), digested with BamHI and XhoI, and ligated into the polylinker of the parent bait plasmid pEG202 as a BamH1-Xho1 fragment. The human* e(r)* coding region was amplified via PCR using the primers 5′ TGGAATTCATGTCTCACACCATTTTG 3′ and 5′ CTCGAGTTATTTCCCAGCCTGTT 3′ (Table 1), digested with EcoRI and XhoI, and ligated into the polylinker of pEG202 as an EcoR1-Xho1 fragment. These procedures fuse the* e(r)* genes in frame with the LexA DNA-binding domain. EGY191/pRB1840 was transformed with either the Drosophila* e(r)* as the bait, pEG202-DER, or the human* e(r)* as the bait, pEG202-HER. Bait proteins expressed from pEG202 will enter the yeast nucleus and bind LexA operators upstream of the two reporter genes* LEU2* and* LACZ* but, alone, will fail to activate their transcription.

### 2.3. Screening the Drosophila and Human cDNA Libraries

A Drosophila 0–12-hour embryo cDNA library was screened for genes encoding ERH-interacting proteins. The library was created in the plasmid pJG4-5 and contains 4 × 10^6^ independent clones [13]. The prey genes in the library consist of a transcription activation domain fused to a random cDNA. If the coding region encodes an ERH-binding protein, then it will bind to LexA-ERH at the LexA promoters of the* LEU2* and* LACZ* genes. This will result in the activation of both genes resulting in a* LEU2* ^+^ *LACZ* ^+^ colony. Another requirement of the screen is that the* LEU2* ^+^ *LACZ* ^+^ phenotypes be galactose-dependent, since the prey genes are under the control of a* Gal1* promoter. The EGY191/pRB1840/pEG202-DER strain was first transformed with the pJG4-5 cDNA library, and a total of 102,000 transformants were isolated. These colonies served as the stock for further screening. Next* LEU2* ^+^ colonies were isolated from the stock on minimal medium containing galactose. A total of 5213 colonies were isolated as putative galactose-dependent* LEU2* ^+^ colonies. Upon retesting, 172 were galactose-dependent. These colonies were then tested for galactose-dependent* LACZ* ^+^ phenotype. Of the 172 colonies, 12 were galactose-dependent* LACZ* ^+^. Finally, the library plasmid from each of these colonies was isolated and retested for its dependence on the bait plasmid, pEG202-DER, for producing the galactose-dependent* LEU2* ^+^ *LACZ* ^+^ phenotype. Eight of these clones proved to be bait-dependent and were saved for further analysis.

A HeLa cell cDNA library was screened to isolate genes encoding human proteins that interact with human ERH. A similar procedure to the Drosophila screen was performed except that pEG202-HER was used. A total of 73,000 individual colonies transformed with the HeLa cDNA library. These produced 18 galactose-dependent* LEU2* ^+^ *LACZ* ^+^ colonies. From these five bait-dependent colonies were identified for further analysis.

### 2.4. Identifying the ERH-Interacting Proteins

Plasmid DNA from the positive clones was isolated and used as the template for PCR. Two pJG4-5 specific primers 5′ CCAGCCTCTTGCTGAGTGGAGATG 3′ and 5′ GACAAGCCGACAACCTTGATTGGA 3′ were used. These primers flank the EcoRI and XhoI sites in pjG4-5 and can be used to amplify the inserted cDNA. The amplified DNA was sequenced and homology searches using BLAST (https://blast.ncbi.nlm.nih.gov/Blast.cgi) were performed to identify the clones.

### 2.5. Coimmunoprecipitation of LexA-DER and HA-RPS3

Yeast expressing LexA-DER and HA-RPS3 was grown in 50 mL minimal galactose medium at 30° with shaking for 48 to 72 hours. To verify the expression of LexA-ERH, one mL of the growth culture was removed to a microcentrifuge tube. The cells were pelleted by centrifugation in a microcentrifuge for 5 min at 12,000 rpm, the supernatant was removed, and the pellet was resuspended in 40 *μ*L Laemmli loading buffer (Bio-Rad Laboratories, Inc.) with *β*-ME. After resuspension, the sample was stored at −20° until being analyzed by SDS-PAGE. The remaining culture was centrifuged at 3000 rpm for 15 min to pellet the cells. The pellet was resuspended in 5 mL CIP buffer (50 mM Tris-HCl pH 7.5, 100 mM NaCl, and 5 mM EDTA 0.1% triton X-100) and protease inhibitor and cooled on ice. A volume of glass beads (Sigma-Aldrich) equal to the volume of the cell pellet was added, and the sample was vortexed 5 times at 30 seconds bursts to shear the cells, with cooling on ice between vortexes. Cell debris was removed from the sample by centrifugation at 3,000 rpm for 5 minutes at 4°. The protein containing supernatant was removed and kept on ice.

A rabbit polyclonal anti-HA antibody (Santa Cruz Biotechnology) was added (1 : 500) to the sample and incubated on ice for 1 hour, with brief mixing in between. 100 *μ*L of protein A beads (Pierce Chemical Co.) was added and allowed to incubate for 1 hour on ice. Brief mixing of the sample was done throughout the 1-hour incubation to keep the beads in suspension. The sample was centrifuged for 3 minutes at 1,500 rpm to pellet the beads and the supernatant was removed. The beads were washed 4 times in 10 mL of CIP buffer. The final bead pellet was resuspended in 40 *μ*L Laemmli loading buffer with *β*-ME and boiled for 1 minute.

As a negative control, yeast expressing LexA and HA-RPS3 was treated in parallel with the sample above. Also, yeast expressing HA and LexA-DER was treated in the same manner to insure DER was not sticking to the protein A beads. 40 *μ*L of each sample was loaded in the order: crude yeast extract expressing LexA/HA-RPS3, yeast extract expressing LexA/HA-RPS3 treated with anti-HA, crude yeast extract expressing LexA-DER/HA-RPS3, yeast extract expressing LexA-DER/HA-RPS3 treated with anti-HA, crude yeast extract expressing LexA-DER/HA, yeast extract expressing LexA-DER/HA treated with anti-HA, and marker.

The samples were run on a 10% polyacrylamide gel (Invitrogen NuPage®, NP0341BOX) in 1x Invitrogen NuPage MES SDS Running Buffer (NP0002) and transferred to a nitrocellulose membrane (Pierce Chemical Co.) using Invitrogen NuPage transfer buffer (NP0006) via electroblotting using a Bio-Rad Trans-Blot SD Semi-Dry Transfer Cell and an EC150 (E-C Apparatus Corporation) power source at 20V for one hour.

After transfer, total protein was detected using a reversible membrane protein staining solution (Pierce Chemical Co.). The membrane was blocked in blocking buffer (5% fat-free powdered milk in TTBS: 9.68 g Tris buffer, 116.96 g NaCl, 1760 *μ*L Tween-20 in 4 L dH_2_O, pH to 7.5) for one hour before probing following standard Western techniques. The membrane was probed with a goat anti-LexA antibody (1 : 1000) in blocking buffer for one hour with gentle shaking. The membrane was rinsed 3 times in TTBS over a one-hour period. The goat anti-LexA antibody was detected with a mouse anti-goat IgG-HRP conjugated secondary antibody (1 : 5000) for one hour with gentle shaking. The membrane was rinsed and the conjugate was detected using SuperSignal® West Femto Substrate (Pierce Chemical Co.) and the blot exposed on film.

### 2.6. Mapping the Interacting Region(s) of ERH

To map the regions of the human and Drosophila ERH that were necessary for interacting with their partners, sets of primers were used to amplify different regions of the coding sequence for ligating to the LexA coding region in pEG202 (Table 1). For the Drosophila ERH these primers were used to amplify coding sequences for amino acids 1–51, 52–104, 1–24, and 25–51. Each amplified sequence was digested with BamHI and XhoI and ligated into pEG202. For the human ERH, coding sequences for amino acids 1–51 and 52–104 were amplified, digested with EcoRI and XhoI, and ligated into pEG202. The newly constructed baits were transformed into EGY191/pRB1840/pJG4-5-RpS3 or EGY191/pRB1840/pJG4-5-RPL19 and tested for the galactose-dependent* LEU2* ^+^ *LACZ* ^+^ phenotype.

### 2.7. Mapping the Nuclear Localization Signal of ERH

GFP-ERH constructs were generated by cloning the human and Drosophila* e(r)* fragments encoding amino acids 1–104, 1–51, and 52–104 and the Drosophila ERH fragments 1–24 and 25–51 into the expression vector pEGFP-C1. Specific primer pairs (Table 1) were used to amplify the sequences, which were digested with BamHI and XhoI and ligated into pEGFP-CI. This procedure fuses the fragments in frame to the C-terminus of GFP. Each plasmid was used to transform SK-HEP cells [14]. SK-HEP cells were grown in EMEM plus 10% calf serum for two days to allow them to adhere to round microscope slide covers. SK-HEP cells were supplied by Dr. Barry Bode, Department of Biology, Saint Louis University (current address Department of Biological Sciences, Northern Illinois University). Prior to transfecting the cells, the transfection mix was prepared as follows: 1 *μ*g DNA was added to 389 *μ*L Eagle's minimal essential medium, EMEM (serum-free), and vortexed. To this mix, 9 *μ*L of TfX™-20 Reagent (Promega Corporation) was added, and the mix was vortexed and allowed to incubate for 10–15 min at room temp. After the incubation period, the growth medium was aspirated off the cells, and the transfection mix was added directly to the cells. The cells were returned to the 37° incubator for 2 hours. At the end of the incubation period, 300 *μ*L EMEM with 10% calf serum was added to the cells, without removing the transfection mix. The cells were returned to the incubator and allowed to grow for another two days before viewing with a fluorescent compound microscope with a GFP filter.

## 3. Results

### 3.1. Identification of Potential Binding Partners of ERH

To identify possible binding partners for Drosophila ERH, a yeast two-hybrid screen [15] was performed using Drosophila LexA-ERH as bait to screen a prey library consisting of Drosophila embryo cDNA clones fused to the transcription activation domain of Gal4. To construct the LexA-ERH fusion gene, PCR was performed to amplify the* e(r)* coding region with restriction sites for cloning into pEG202 (Table 1). Positive prey colonies were seen as galactose-dependent* LEU2* ^+^ *LACZ* ^+^ colonies. Eight positive colonies out of 102,000 transformants were isolated. The prey plasmid from each positive clone was isolated and a partial sequence of the cDNA insert was determined to identify the putative ERH-binding partner. Five of the clones were of RPS3 (ribosomal protein S3) and three of the clones were of RPL19 (ribosomal protein L19).

Another yeast two-hybrid screen was performed using the human ERH as bait and a human HeLa cell cDNA prey library. Five positive colonies were identified out of 73,000 transformants. Three of the clones were human RPS3 and two were human DDIT4 (DNA damage inducible transcript 4).

All of the putative binding partners of DmERH and HsERH are known nuclear proteins, which is consistent with previous studies that localized ERH to the nucleus [6, 12]. The fact that RPS3 was identified as a binding partner of ERH in both Drosophila and humans suggests a conserved function of ERH in both humans and Drosophila.

### 3.2. Verification of the Binding of ERH and RPS3 via Coimmunoprecipitation

**Figure 2:**
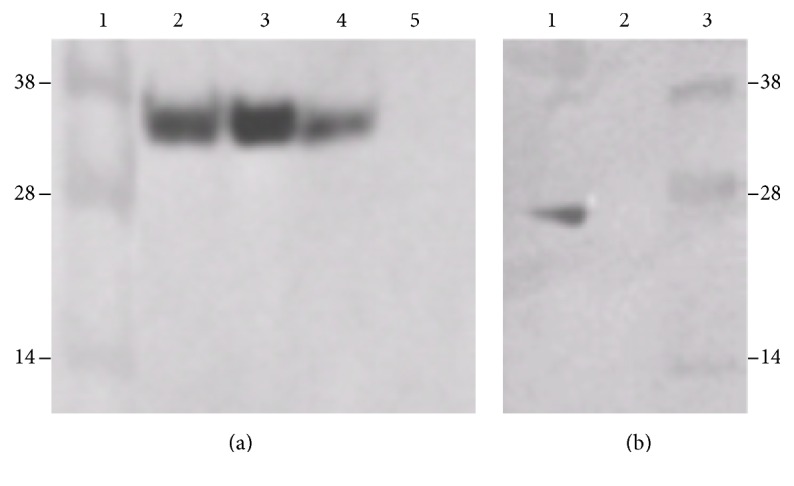
Western blots probed with anti-LexA, showing that ERH and RPS3 bind to each other and can be coimmunoprecipitated. (a) Requirement of RPS3 for coimmunoprecipitation. This blot shows that RPS3 must be attached to HA in order for the HA antibody to coprecipitate LexA-ERH. (1) Molecular weight markers. These were superimposed from a photograph of the stained gel. (2) Extracts from cells expressing HA-RPS3 and LexA-ERH. (3) Immunoprecipitates of extracts from cells expressing HA-RPS3 and LexA-ERH immunoprecipitated with anti-HA antibody. (4) Extracts from cells expressing HA and LexA-ERH. (5) Immunoprecipitates of extracts from cells expressing HA and LexA-ERH that have been immunoprecipitated with anti-HA antibody. (b) Requirement of ERH for coimmunoprecipitation. This blot shows that ERH must be attached to LexA in order for the HA antibody to coprecipitate LexA and rules out the possibility that RPR3 binds to LexA alone. (1) Extracts from cells expressing HA-RPS3 and LexA. (2) Immunoprecipitates of extracts from cells expressing HA-RPS3 and LexA and immunoprecipitated with anti-HA antibody. (3) Molecular weight markers. These were superimposed from a photograph of the stained gel. Together the two blots show that both RPS3 and ERH are necessary to get coimmunoprecipitation and confirm that RPS3 and ERH are binding partners.

The yeast two-hybrid screen is a genetic test to show protein-protein interactions. To verify that Drosophila ERH and RPS3 actually bind to each other, experiments to show coimmunoprecipitation of ERH and RPS3 were performed. The first experiment was designed to show the requirement for the presence of RPS3 for the precipitation of ERH. Yeast cells were grown which expressed LexA-ERH and either HA or HA-RPS3. Protein extracts from each cell type were isolated and immunoprecipitated with anti-HA. LexA-ERH was not present in the immunoprecipitate from the cells expressing HA (Figure 2(a), lane 5) but was present in the immunoprecipitate from the cells expressing HA-RPS3 (Figure 2(a), lane 3). Controls showed that both cell types expressed LexA-ERH (Figure 2(a), lanes 2 and 4). While these experiments showed that RPS3 is necessary for the immunoprecipitation of LexA-ERH, RPS3 could conceivably bind to either LexA or ERH. To show that RPS3 was not binding to LexA, a protein extract was isolated from cells expressing HA-RPS3 and LexA. The extracts were precipitated with anti-HA. In this case LexA did not coprecipitate, demonstrating the necessity for ERH in the coimmunoprecipitation (Figure 2(b), lane 2).

### 3.3. Mapping Interacting Portion of DmERH and HsERH with RPS3 and RPL19

Structural studies on purified human ERH reveal that the protein likely forms a single domain with three *α*-helices and one *β*-sheet composed of four antiparallel *β*-strands [3, 4]. The crystal structure also suggests that the *β*-sheet serves as a surface for protein-protein binding. Given this structural information, it was of interest to determine the amino-acid sequences of ERH that are necessary for its binding to its partners, RPS3 and RPL19. The yeast two-hybrid interaction assay was used to map the ERH sequences necessary for binding. PCR was used to amplify specific regions of the ERH coding region, which were then ligated into the LexA bait plasmid, pEG202 (Table 1). In this way, LexA-ERH baits containing Drosophila ERH amino acids 1–51, 52–104, 1–24, and 25–51 were generated and tested for their ability to interact with RPS3 and RPL19. The results are summarized in Table 2. As a positive control, the full-length ERH (1–104) was shown to bind to RPS3. It also binds to the human RPS3, indicating that this protein-protein interaction is conserved. Amino acids 1–51 were shown to bind to RPS3 and RPL19, whereas amino acids 52–104 did not. Amino acids 1–51 were then subdivided into 1–24 and 25–51. Amino acids 1–24 bind to RPS3 and RPL19, while amino acids 25–51 do not. This maps the sequences necessary for binding to amino acids 1–24.

A similar mapping experiment was performed with HsERH and HsRPS3. In these experiments the human ERH was subdivided into amino acids 1–51 and 52–104 and examined for their ability to bind to HsRPS3. Similar to the results with DmERH, amino acids 1–51 bind to HsRPS3, while amino acids 52–104 do not (Table 3).

### 3.4. Mapping ERH Sequences Necessary for Nuclear Localization

**Figure 3:**
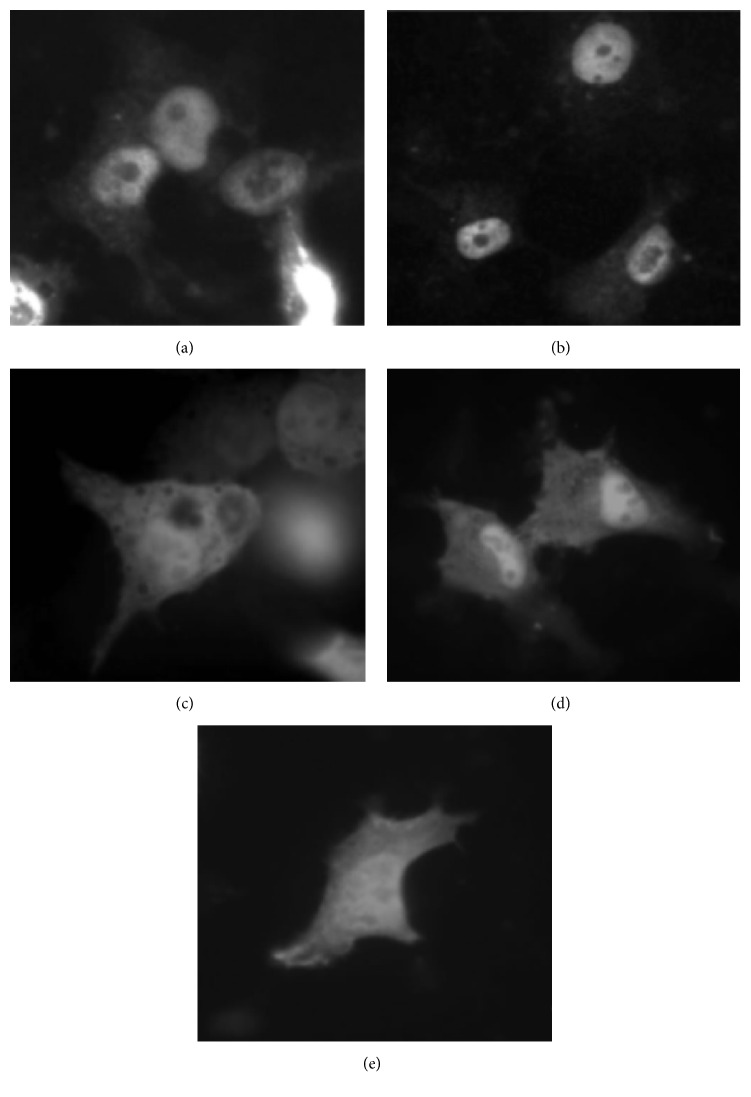
Drosophila ERH sequences necessary for nuclear localization. The cellular localization of fusion proteins containing fragments of Drosophila ERH fused to EGFP was examined in transfected human SK-HEP cells. These experiments show that amino acids 1–24 are necessary and sufficient to localize EGFP to the nucleus. (a) The entire protein, amino acids 1–104, fused to EGFP. (b) Amino acids 1–51 fused to EGFP. (c) Amino acids 52–104 fused to EGFP. (d) Amino acids 1–24 fused to EGFP. (e) Amino acids 25–51 fused to EGFP.

**Figure 4:**
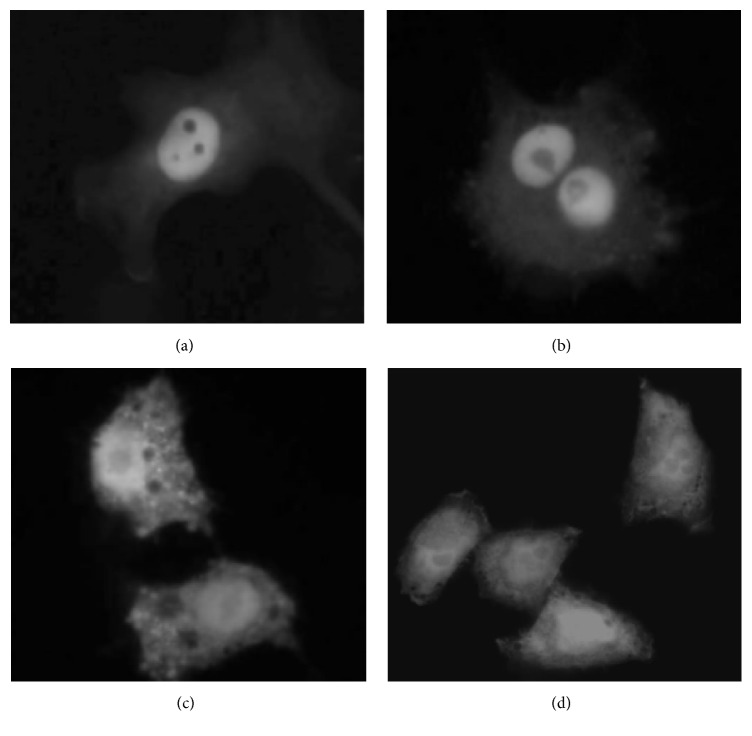
Human ERH sequences necessary for nuclear localization. The cellular localization of fusion proteins containing fragments of human ERH fused to EGFP was examined in transfected human SK-HEP cells. These experiments show that amino acids 1–51 are necessary and sufficient to localize EGFP to the nucleus. (a) The entire protein, amino acids 1–104, fused to EGFP. (b) Amino acids 1–51 fused to EGFP. (c) Amino acids 52–104 fused to EGFP. (d) EGFP by itself.

Previous studies on the immunolocalization of ERH in Drosophila S2 cells and early embryos demonstrated that in* Drosophila melanogaster* endogenous ERH is nuclearly localized [12]. Those results corroborate studies in human cell lines in which EGFP-HsERH fusion proteins were localized to the nucleus [6]. These data argue that ERH must have a nuclear localization signal; however, scans of the Drosophila and human ERH amino-acid sequences using PredictProtein (http://PredictProtein.org/), WoLF PSORT (http://www.genscript.com/wolf-psort.html/), and cNLS mapper (http://nls-mapper.iab.keio.ac.jp/cgi-bin/NLS_Mapper_form.cgi/) have not revealed any nuclear localization signals. This leaves unanswered the questions as to how ERH is nuclearly localized and what amino-acid sequences are necessary for nuclear localization. We decided to utilize the GFP system to identify the sequences necessary for the nuclear localization of ERH. Both Drosophila ERH and human ERH were utilized in the study. Human SK-HEP cells were used as the host cells in the assay. PCR was used to amplify all or part of the coding regions for ligation into the plasmid, pEGFP-C1, for the creation of GFP-DmERH and GFP-HsERH fusions (Table 1). These fusions result in appending ERH fragments onto the C-terminus of EGFP. Plasmids containing the fusion genes were transfected into human SK-HEP cells and cellular localization was visualized with GFP. The results are summarized in Table 4. For both ERH and HERH, the full-length protein localizes to the nucleus (Figures 3(a) and 4(a)). The N-terminal half of each protein (amino acids 1–51) can also direct nuclear localization (Figures 3(b) and 4(b)), while the C-terminal half of each protein (amino acids 52–104) does not (Figures 3(c) and 4(c)). For Drosophila ERH, the N-terminal half was further subdivided into amino acids 1–24 and 25–51. Amino acids 1–24 direct the localization of GFP to the nucleus (Figure 3(d)), although there appeared to be more cytoplasmic expression of GFP than what was seen when amino acids 1–51 (Figure 3(b)) or the entire protein (Figure 3(a)) was used. Amino acids 25–51 fail to nuclearly localize GFP (Figure 3(e)). These data localize the minimal sequence needed for nuclear localization to amino acids 1–24. As a negative control, EGFP by itself does not nuclearly localize (Figure 4(d)).

## 4. Discussion

The data argue that the first 24 amino acids are sufficient for the nuclear localization of ERH. There are two arginines within this region of Drosophila ERH, but nothing looks like a nuclear localization signal. Interestingly, this 24-amino-acid region is the same region that is necessary for ERH to bind to its binding partners, RPS3 and RPL19 (Table 2). Both of these proteins contain nuclear localization signals, as identified by cNLS mapper. The Drosophila RPS3 in particular has a strong NLS starting at amino acid 6 (PIS$\underline{\underline{KKRK}}$FV). The human RPS3 has a similar NLS starting at amino acid 4 ($QIS\underline{\underline{KKRK}}FV$). In addition, RPS3 has been shown to localize to the nucleus in both* Drosophila melanogaster* [16] and humans [17]. Together these data suggest that ERH may be entering the nucleus through piggy backing onto one of its binding partners, such as RPS3.

The N-terminal 24-amino-acid region, which is necessary for both the nuclear localization of ERH and its binding to RPS3 and RPL19, is comprised of two antiparallel *β*-strands that are part of a four-strand *β*-sheet. This *β*-sheet serves as a hydrophobic surface in protein binding [3, 4]. Five out of the seven residues within the *β*-sheet, which have been identified as important in the dimerization of ERH, are contained within the first two *β*-strands. These amino acids are 5I, 7L, 17R, 19Y, and 21D. These residues are conserved between human and Drosophila ERH (Figure 1), suggesting their importance in protein binding in both species, and may explain how Drosophila ERH can bind to both Drosophila and human RPS3. Our current results also indicate that while the four strands of the *β*-sheet form a face for protein binding, the first two *β*-strands are necessary and sufficient for protein binding and nuclear localization. Although amino acids 1–24 are sufficient for nuclear localization, this sequence appears not to localize GFP to the nucleus as strongly as do amino acids 1–52 and 1–104 (Figure 3). These additional amino acids (25–104) are not sufficient for nuclear localization, but they may help stabilize the structure of amino acids 1–24 for optimal protein binding. Amino acids 25–52 comprise a major *α*-helix that is positioned behind the two *β*-strands of amino acids 1–24. This structure may aid in presenting the two *β*-strands to ERH's binding partners. Amino acids 66–82 comprise the last two *β*-strands that, along with amino acids 1–24, form the *β*-sheet that is necessary for the binding of ERH to its partners. These *β*-strands, while helping to stabilize the *β*-sheet, also contain two amino acids that have been shown to be contact points in protein-protein binding of ERH. These amino acids should strengthen the interactions and thus strengthen the nuclear localization of ERH.

The present studies identified three new putative binding partners for ERH, RPS3, and RPL19 in Drosophila and RPS3 and DDIT4 in humans. There are some common themes in the functions of these proteins. RPS3, besides being a ribosomal protein, is a DNA repair enzyme [16, 18] and DDIT4 is a protein whose expression is induced in response to DNA damage [19]. Also, human RPS3 has been shown to translocate from the cytoplasm to the nucleus in response to genotoxic stress treatment with hydrogen peroxide or DNA alkylating agents [20]. These findings suggest the possibility that ERH has a role in DNA damage repair and that this function can be regulated by regulating its nuclear localization through its binding to RPS3.

There is a high evolutionary conservation between the human and Drosophila ERH. Both are 104 amino acids in length and can be lined up without gaps along their entire lengths [2]. Additionally, the two proteins show 76% amino acid identity and 84% similarity [2]. The tertiary structure or the Drosophila ERH [21], predicted by SWISS-MODEL [22], is nearly identical to the solved tertiary structure of the human/mouse ERH [3, 4]. For the human/mouse ERH, seven amino acids were identified as critical in protein binding (5I, 7L, 17R, 19Y, 21D, 70L, and 79Y). Six of these seven amino acids are strictly conserved in the Drosophila ERH [21]. The one difference is a conservation amino acid substitution at residue 70 (M for L). The first five of these amino acids are contained within the N-terminal 24 amino acids that this study has shown to be important for both binding of ERH to RPS3 and RPL19 and the nuclear localization of ERH. This high structural conservation suggests that the human and Drosophila ERH may be functionally equivalent. Recently, this has been demonstrated through transgenic studies [21]. In these studies, the Drosophila ERH coding region was replaced with the human ERH coding region in the Drosophila genome without any noticeable effect on the fly. All of the known mutant phenotypes associated with the deletion of the Drosophila* e(r)* gene were rescued by the human* e(r)* gene, indicating that the human ERH can functionally replace the Drosophila ERH.

The evolutionary conservation in the structure and function of the human and Drosophila ERH suggest that its roles in the cell may also be conserved between humans and Drosophila. Given this possibility, the fact that the present study identified RPS3 as a possible binding partner for both the human and Drosophila ERH is intriguing. Like ERH, human and Drosophila RPS3 are structurally and functionally very similar. They share an 83% amino acid identity and an 89% amino acid similarity [23] and both have been shown to be DNA repair proteins [16, 18]. It may be that the roles and mechanisms of ERH and RPS3 in growth and DNA repair are conserved between humans and Drosophila.

RPL19 and RPS3 are both upregulated in high growth situations, specifically in certain cancer cells [24–27]. RPS3 has also been shown to be a transcription factor. As a subunit of NF-*κ*B, it is responsible for the activation of specific NF-*κ*B target genes [17]. These nuclear functions, along with previously identified nuclear functions, implicate ERH in transcription initiation, transcription elongation, mRNA splicing, DNA synthesis, and DNA repair. ERH is a very small protein and to date the only biochemical function that has been attributed to it is protein binding. It may be that ERH is modulating the activity of a number of different nuclear complexes purely through binding to specific subunits of the complexes.

## 5. Conclusions

Yeast two-hybrid interaction trap assays identified RPS3, RPL19, and DDIT4 as binding partners of ERH. Similarities in the function of these proteins suggest a role of ERH in DNA repair and cell growth. Amino acids 1–24 of ERH were shown to be sufficient and necessary for the binding to RPS3 and RPL19. This region was also shown to be sufficient for the nuclear localization of ERH. The role of this region in these two properties of ERH suggests a model for its nuclear localization. ERH, which appears not to have an NLS, may be localizing to the nucleus via binding to one of its partners which has an NLS.

## Figures and Tables

**Table 1:** Primers used in the construction of *e(r)* fusion genes.

Species	Target	Primer sequences 5′ to 3′	Vector	Restriction site
H.s.	ERH 1 5′	TGGAATTC $\underline{\underline{ATG}}$TCTCACACCATTTTG	pEG202	EcoRI
H.s.	ERH 52 5′	TGGAATTC $\underline{\underline{TAT}}$GACATCAGTCAGTTGTTT	pEG202	EcoRI
H.s.	ERH 51 3′	TGCTCGAG *TTA* $\underline{\underline{TGT}}$GATAGAGGGACT	pEG202	XhoI
H.s.	ERH 104 3'	GGCTCGAG *TTA* $\underline{\underline{TTT}}$CCCAGCCTGTT	pEG202	XhoI

D.m.	ERH 1 5′	GGGGATCCCC$\underline{\underline{ATG}}$TCGCACACCATCCT	pEG202	BamHI
D.m.	ERH 25 5′	GGGGATCCCC$\underline{\underline{GTC}}$AACGAGTGCATGGG	pEG202	BamHI
D.m.	ERH 52 5′	GGGGATCCCC$\underline{\underline{TAT}}$GACATTAGCCAGCTATTC	pEG202	BamHI
D.m.	ERH 24 3′	CCCTCGAG *TTA* $\underline{\underline{GCT}}$CTCGTAGTCACAGTA	pEG202	XhoI
D.m.	ERH 51 3′	CCCTCGAG *TTA* $\underline{\underline{CGT}}$AATGGTCGGAGTGTT	pEG202	XhoI
D.m.	ERH 104 3′	GGCTCGAG *TTA* $\underline{\underline{GGT}}$ATTGGAACTAA	pEG202	XhoI

H.s.	ERH 1 5′	AGATCTCGAGCT$\underline{\underline{ATG}}$TCTCACACCATTTTGCTGG	pEGFP-C1	XhoI
H.s.	ERH 52 5′	TGCTCGAGCT$\underline{\underline{TAT}}$GACATCAGTCAGTTGTTT	pEGFP-C1	XhoI
H.s.	ERH 51 3′	TGGGATCC *TTA* $\underline{\underline{TGT}}$GATAGAGGGACT	pEGFP-C1	BamHI
H.s.	ERH 104 3′	GGTTTCGGATCCCGATTGAACAAGATCCTCAC	pEGFP-C1	BamHI
		*e(r)* sequence starts within the 3′ UTR.		

D.m.	ERH 1 5′	GGCTCGAGCT$\underline{\underline{ATG}}$TCGCACACCATCCT	pEGFP-C1	XhoI
D.m.	ERH 25 5′	GGCTCGAGCT$\underline{\underline{GTC}}$AACGAGTGCATGGAGGG	pEGFP-C1	XhoI
D.m.	ERH 52 5′	GGCTCGAGCT$\underline{\underline{TAT}}$GACATTAGCCAGCTATTC	pEGFP-C1	XhoI
D.m.	ERH 24 3′	CCGGATCC *TTA* $\underline{\underline{GCT}}$CTCGTAGTCACAGTA	pEGFP-C1	BamHI
D.m.	ERH 51 3′	CCGGATCC *TTA* $\underline{\underline{CGT}}$AATGGTCGGAGTGTT	pEGFP-C1	BamHI
D.m.	ERH 104 3′	CCGGATCC *TTA* $\underline{\underline{GGT}}$ATTGGAACTAA	pEGFP-C1	BamHI

The target indicates the amino-acid codon that starts the *e(r)* sequence in the primer and the position of the primer. In the primer sequence, the restriction site is underlined and the starting codon is double underlined and in bold. For the 3′ primers, the stop codon is italicized. For the H.s. ERH 104 3′ for pEGFP-C1 the primer starts in the 3′ UTR, so there is not a stop codon in this primer. D.m., *Drosophila melanogaster*; H.s., *Homo sapiens*.

**Table 2:** Drosophila ERH interactions with its partners.

ERH bait segment	Prey protein	Interaction
1–104	D.m. RPS3	+
1–104	H.s. RPS3	+
1–104	D.m. RPL19	+

1–51	D.m. RPS3	+

1–24	D.m. RPS3	+
1–24	D.m. RPL19	+

52–104	D.m. RPS3	−

25–51	D.m. RPS3	−

The table summarizes the bait-prey interactions performed in the yeast two-hybrid interaction assay. The amino acid segments of the ERH baits are given. A + interaction indicates that yeast containing a prey and the bait exhibits both the galactose-dependent growth on medium lacking leucine (*LEU* ^+^) and the galactose-dependent beta-galactosidase activity (*LACZ* ^+^) on medium containing X-gal. A − interaction indicates that the yeast failed both of the galactose-dependent assays. D.m., *Drosophila melanogaster*; H.s., *Homo sapiens*.

**Table 3:** Human ERH interactions with its partners.

ERH bait segment	Prey protein	Interaction
1–104	H.s. RPS3	+
1–104	H.s. DDIT4	+

1–51	H.s. RPS3	+

52–104	H.s. RPS3	−

The table summarizes the bait-prey interactions performed in the yeast two-hybrid interaction assay. The amino acid segments of the ERH baits are given. A + interaction indicates that yeast containing a prey and the bait exhibits both the galactose-dependent growth on medium lacking leucine (*LEU* ^+^) and the galactose-dependent beta-galactosidase activity (*LACZ* ^+^) on medium containing X-gal. A − interaction indicates that the yeast failed both of the galactose-dependent assays. H.s., *Homo sapiens*.

**Table 4:** EGFP-ERH nuclear localization in human SK-HEP cells.

Species	ERH segment	Nuclear
D.m.	1–104	+
	1–51	+
	1–24	+
	52–104	−
	25–51	−

H.s.	1–104	+
	1–51	+
	52–104	−

The cellular localization of the EGFP-ERH fusion proteins is given. A strong nuclear localization is given a positive score. Both a nuclear and cytoplasmic localization similar to that of EGFP alone is given a negative score. The evaluations were made from the microscopic examinations of EGFP localization, examples of which are shown in Figures 3 and 4. D.m., *Drosophila melanogaster*; H.s., *Homo sapiens*.
